# Prognosis and delay of diagnosis among Kaposi’s sarcoma patients in Uganda: a cross-sectional study

**DOI:** 10.1186/1750-9378-9-17

**Published:** 2014-05-20

**Authors:** Christopher De Boer, Nixon Niyonzima, Jackson Orem, John Bartlett, S Yousuf Zafar

**Affiliations:** 1 Duke Global Health Institute, Duke University, 310 Trent Drive, Durham, NC 27710, USA; 2 Uganda Cancer Institute, Upper Mulago Hill Rd, Kampala, Uganda; 3 Duke University Medical Center, Duke University, 10 Bryan Searle Drive, Durham, NC 27710, USA; 4 College of Health Sciences, Makerere University, Kampala, Uganda; 5 Uganda Program on Cancer and Infectious Disease, Kampala, Uganda; 6 Duke Cancer Institute, Durham, NC, USA; 7 University of Washington, Seattle, WA, USA; 8 Fred Hutchinson Cancer Research Center, Seattle, WA, USA; 9 University of Iowa Carver College of Medicine, 375 Newton Road, Iowa City, IA 52242, USA

**Keywords:** Kaposi’s sarcoma, Delayed diagnosis, HIV, Uganda, Cancer

## Abstract

**Background:**

In low- and middle-income countries, the association between delay to treatment and prognosis for Kaposi’s sarcoma (KS) patients is yet to be studied.

**Methods:**

This is a prospective study of HIV-infected adults with histologically-confirmed KS treated at the Uganda Cancer Institute (UCI). Standardized interviews were conducted in English or Luganda. Medical records were abstracted for KS stage at admission to UCI. Multivariable logistic regression assessed relationships between diagnostic delay and stage at diagnosis.

**Results:**

Of 161 patients (90% response rate), 69% were men, and the mean age was 34.0 years (SD 7.7). 26% had been seen in an HIV clinic within 3 months, 72% were on antiretroviral therapy, and 26% had visited a traditional healer prior to diagnosis. 45% delayed seeking care at UCI for ≥3 months from symptom onset. Among those who delayed, 36% waited 6 months, and 25% waited 12 months. Common reasons for delay were lack of pain (48%), no money (32%), and distance to UCI (8%). In adjusted analysis patients who experienced diagnostic delay were more likely than those who did not delay to have poor-risk KS stage (OR 3.41, p = 0.002, 95% CI: 1.46-7.45). In adjusted analyses visiting a traditional healer was the only variable associated with greater likelihood of delay (OR 2.69, p = 0.020, 95% CI: 1.17-6.17).

**Conclusions:**

Diagnostic delay was associated with poor-risk stage at diagnosis, and visiting a traditional healer was associated with higher odds of delay. The relationship between traditional and Western medicine presents a critical intervention point to improve KS-related outcomes in Uganda.

## Background

The incidence of Kaposi’s sarcoma (KS) in sub-Saharan Africa has increased since the advent of the HIV epidemic, contributing an estimated 37,214 cases and 25,352 deaths annually [1]. While incidence and mortality have decreased in high-income countries with the introduction of antiretroviral therapy (ART), low-income countries have seen the opposite, especially those in sub-Saharan Africa that carry heavy burdens of HIV. Over the same period in which the West saw a 24-fold decrease in incident KS, Uganda and Zimbabwe experienced a 20-fold increase in KS incidence among HIV-infected individuals [2,3]. This has been attributed to the rise of HIV/AIDS and the lack of antiretroviral therapy, which has been shown to be crucial for KS tumor regression, decreasing viral loads, and raising CD4 counts. [4-9]. Because of these factors, KS is now the most common cancer among HIV-infected men in SSA and the second most common in women after cervical cancer [1].

In Uganda, KS contributes 3,635 annual incident cases with 2,637 annual deaths [1]. Previous research has pointed to a lack of coverage with ART, gender differences, and high seroprevalence of human herpesvirus type 8 (HHV-8), the causative agent of KS, to explain poor patient outcomes in Uganda [9-11]. ART coverage in the country remains below 50 percent, and persistent KS has been noticed despite prompt treatment with ART [12]. Other patients have experienced immune reconstitution inflammatory syndrome (IRIS) after the initiation of ART, causing a proliferation of KS [13]. While these explanations offer some insight into the poor patient outcomes for KS patients in Uganda, numerous factors remain unexamined, particularly those concerning the health system and access to cancer care for KS patients.

Diagnostic delay has been assessed as a health system factor that can influence cancer stage and patient prognosis among specific cancers in developed countries [13-17]. One type of delay that has been shown to be associated with poor patient prognoses is “primary” delay, defined as waiting longer than three months after noticing signs and symptoms before presentation to a clinician [18-20]. Other types of delay are traditionally defined as secondary delay, the time from presentation to a clinician until diagnosis, and tertiary delay, the time from diagnosis to initiation of treatment [18]. A search of the literature did not reveal any known associations between KS stage with diagnostic delay at any stage in low- and middle-income countries (LMICs). Further, we could not find an analysis of this association with respect to HIV-associated malignancies in any context. Therefore, the primary aim of this study was to measure the association between primary delay and the cancer stage of HIV-KS patients upon diagnosis of the disease by a clinician. We hypothesized that those who experienced primary delay would be more likely to present with late-stage KS than those who did not delay.

## Materials and methods

### Study design

This was a cross-sectional, prospective study to measure the association between diagnostic delay, specifically primary delay greater than three months, and having an overall poor HIV-associated KS stage risk. Staging data were abstracted from chart records, and history of diagnostic delay was gathered through standardized interviews. Patients who reported waiting longer than three months after noticing signs and symptoms before reporting this to any health professional were defined as “delayers”. The cutoff of three months was established based on previous literature examining delay within cancer populations that has utilized and validated the same value [14,18-20]. Medical record data and standardized interviews completed in English and Luganda were utilized to obtain data on the history of exposure to ART, presence of primary, secondary, and tertiary delay, and patient demographics. A cutoff of three weeks was established for secondary and tertiary delay, which was the suggested value from clinicians at the UCI familiar with the local health system and the patient population.

We utilized the prospectively validated staging system for AIDS-associated Kaposi sarcoma as designed by the AIDS Clinical Trials Group (ACTG), which is used by physicians at the UCI [21]. Patients were dichotomized as either having overall “good risk” or “poor risk” based on this system, which designates good or poor risk in three diagnostic areas: extent of tumor involvement (T), immune system function (I), and presence of systemic illness (S). A patient with poor risk in all three areas, (T_1_I_1_S_1_), is defined as having overall poor risk as their HIV-KS stage. Poor risk for extent of tumor involvement is described as having any tumor-associated edema, extensive raised KS, oral KS nodules not confined to the palate, or any KS of the gastrointestinal tract or any other non-nodal viscera. Good risk for tumor extent indicates that all KS nodules, or lesions, are confined to the skin or lymph nodes, and any oral involvement is confined to the palate only. Poor risk for immune system function is defined as any patient that has a CD4 count that is ≤ 150 cells per cubic microliter as per the modification to the staging system made by the ACTG and implemented at the UCI [22]. For presence of systemic illness, poor risk is defined as having any history of opportunistic infections, or the presence of “B” symptoms, or any other HIV-related illness (e.g. neurologic disease, lymphoma). B symptoms are defined as the presence of HIV-associated illness by the CDC Clinical Category B, which includes drenching night sweats, greater than 10% body weight loss, unexplained fevers, or diarrhea persisting for greater than two weeks.

The ACTG staging system has been prospectively validated to link stage and survival in both resource rich and resource limited contexts [22,23]. In a high-income setting, survival was significantly shorter for patients that displayed poor risk in each diagnostic category with the Immune status (I) and Tumor extent (T) categories being most predictive of survival, but Systemic symptoms (S) was not [22]. In a resource limited setting, however, the T and S categories were associated with survival, but the I diagnostic category was only associated with survival when establishing a cutoff of CD4 counts at <100 cells/microliter [23]. The authors of that study concluded that more research is needed to examine the implementation of the ACTG staging system in populations in sub-Saharan Africa given different prognostic factors. This study represents an opportunity to implement the system in such a setting to see if delay is a prognostic factor that influences ACTG stage among KS patients upon admission.

### Study population

Eligible participants were HIV seropositive out-patients with histologically confirmed AIDS-associated Kaposi’s sarcoma. The study was performed at the Uganda Cancer Institute (UCI) in Kampala, Uganda, the only specialized cancer treatment center in the country, from June-October 2012. Although the UCI provides treatment for free for KS patients, transportation and other costs associated with care are considerable challenges for many patients. The study was performed among adults (≥18 years old) who had histologically confirmed Kaposi sarcoma, were HIV seropositive, and had a CD4 count performed within the previous six months. Patients with a history of immune reconstitution inflammatory syndrome (IRIS), who would be severely ill, were excluded from the study, although we did not encounter any of these individuals through the interview process, most likely due to our focus on the out-patient population.

### Statistical analysis

Descriptive statistics were measured using means with standard deviations and percentages to obtain prevalence of delay, mean ages, age distribution, and prevalence of different staging criteria. Pearson’s Chi-Square test was utilized for unadjusted analyses. Multivariate logistic regression with a generalized linear model assumption with binary outcomes was implemented to model all adjusted associations and obtain prevalence odds ratios. A multivariate model was created to measure the association between primary delay and an overall poor stage risk, adjusting for age, gender, ability to pay out-of-pocket, income, and exposure to ART. Gender was included in the model because it was significantly associated with overall poor stage risk in the univariate analysis (Table 1). Age, income, and exposure to ART were anchored in the model based on clinical knowledge and previous literature that suggested their association with delay and cancer prognosis [10,11].

**Table 1:** Patient characteristics by patient status of overall stage risk

**Characteristic**	**Overall stage risk**						
	**Good risk**		**Poor risk**		**Total**		P-Value*
	**n**	**%**	**N**	**%**	**N**	**%**	
**Patient gender**							
Male	74	64.3	37	80.4	111	68.9	
Female	41	35.7	9	19.6	50	31.1	0.046
Total	115	100.0	46	100.0	161	100.0	
**Patient age**							
<30	35	31.3	9	20.0	44	28.0	
31-40	59	52.7	26	57.8	85	54.1	
>40	18	16.1	10	22.2	28	17.8	0.319
Total	112	100.0	45	100.0	157	100.0	
**Monthly income**							
<100,000 UGSH	63	57.3	27	60.0	90	58.1	
100K-500K UGSH	45	40.9	14	31.1	59	38.1	
>500,000	2	1.8	4	8.9	6	3.9	0.083
Total	110	100.0	45	100.0	155	100.0	
**Level of education**							
Primary	57	49.6	26	56.5	83	51.6	
Secondary	44	38.3	14	30.4	58	36.0	
Tertiary or Degree	14	12.2	6	13.0	20	12.4	0.642
Total	115	100.0	46	100.0	161	100.0	
**Paid out-of-pocket for tests or chemo**							
No	57	49.6	36	78.3	93	57.8	
Yes	58	50.4	10	21.7	68	42.2	0.001
Total	115	100.0	46	100.0	161	100.0	
**Visited traditional healer**							
Yes	30	26.1	11	23.9	41	25.5	
No	85	73.9	35	76.1	120	74.5	0.872
Total	115	100.0	46	100.0	161	100.0	
**Exposure to ART**							
No	27	23.5	17	37.0	44	28.4	
Yes	82	71.3	29	63.0	111	71.6	0.124
Total	115	100.0	46	100.0	155	100.0	
**Primary delay**							
< 3 months	73	63.5	15	32.6	88	54.7	
> = 3 months	42	36.6	31	67.4	73	45.3	
Total	115	100.0	46	100.0	161	100.0	<0.001

*Unadjusted, using Pearson’s Chi-Square test.

Of note, ability to pay out-of-pocket, a dichotomous variable referring to a patient’s ability to pay any cash out of pocket for any previous KS treatment before presentation to UCI, was significantly associated with overall poor stage risk in unadjusted analysis (Table 1). We determined that this variable was a strong modifier and important protective factor in the relationship between primary delay and overall stage risk as a proxy for a patient’s wealth but not an independent determinant of overall poor stage risk.

Characteristics that were significantly associated with primary delayers in unadjusted analyses were implemented in a second multivariate model to measure its association with primary delay, adjusting for age, gender, income, ability to pay-out-of pocket, and exposure to ART. These covariates were anchored in the model based on clinical knowledge and previous literature that suggested they may be associated with the variables of interest [10-12]. Data were stored in Microsoft Excel 2010 and analyzed using Stata/SE v.11.0 (College Station, Texas).

### Funding source and ethical approval

This study was funded by the Master of Science in Global Health Student Research Grant from the Duke Global Health Institute in Durham, NC. Ethical approval was provided by Duke University Institutional Review Board for Research with Human Subjects and the Makerere College of Health Sciences Research Ethics Committee.

## Results

From June 22 to October 30, 2012, 178 consecutive AIDS-associated KS patients treated at the Uganda Cancer Institute as out-patients were approached for participation; 168 agreed to participate and 7 surveys were discarded due to data quality issues (90% response rate). Sixty-nine percent were men, and the mean age was 34 years (SD: 7.7) with a slightly lower age distribution for women, although the mean age was not significantly different between men and women (p = 0.13). Fifty-eight (35%) of all participants were unemployed, and 49 percent had at least a primary education. Among all participants, 149 (93%) had previously received some form of treatment at an HIV clinic upon admission to UCI, and 106 (72%) of all participants were taking antiretroviral therapy (ART). Of those, 77 had been exposed to ART for at least three months prior to enrollment. Twenty-six percent of all participants reported visiting a traditional healer prior to their admission to UCI (Table 1).

**Figure 1:**
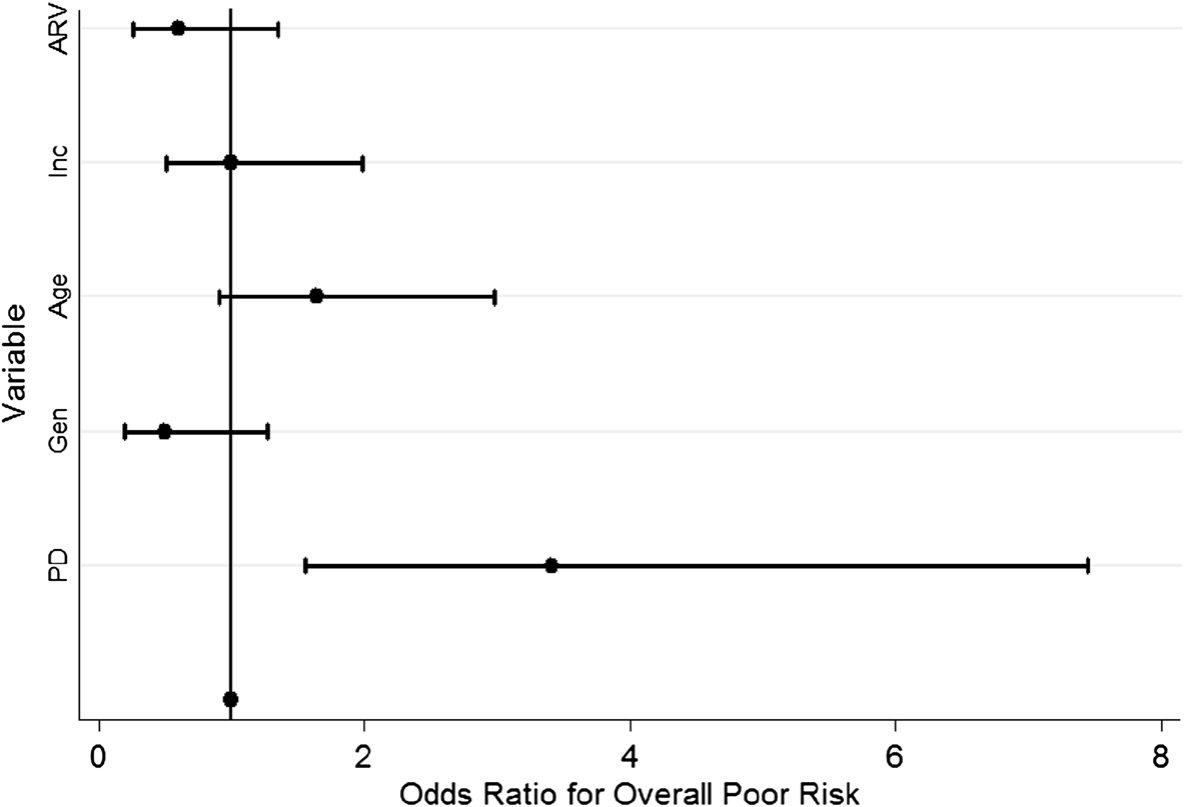
Plot of Odds Ratios for the association between primary delay and overall poor stage risk with selected covariates.

Among all patients, 73 (45%) experienced primary diagnostic delay longer than three months (Table 2). Among those who delayed, 26 (36%) waited for more than 6 months, while 18 (25%) waited for more than 12 months prior to seeking medical attention for KS-related symptoms. Lack of pain (48%), lack of money for transportation (32%), and distance to UCI (8%) were cited as the most common reasons for delay. In addition, 46 (29%) had an overall poor risk as their KS stage upon admission (Table 2). Ninety-two percent of all patients presented with poor risk in the tumor extent category (T), 40% of patients had a CD4 count less than 150 cells/microliter, categorizing them as poor risk with respect to immune system function (I), and 73% had poor risk with respect to presence of systemic illness (S). In multivariate analysis after adjusting for gender, age, income, and exposure to ART, patients who experienced diagnostic delay were more than three times as likely to have poor-risk stage at presentation compared to those who did not delay (OR 3.41, p = 0.002, 95%CI: 1.46-7.45) (Figure 1, Table 3).

**Table 2:** Primary findings: distribution of primary delay and overall stage risk (n = 161)

	**Total**	
	**N**	**%**
**Primary delay**		
<3 Months	88	54.7
≥ 3 Months	73	45.3
**Overall stage risk**		
Good risk	115	71.4
Poor risk	46	28.6

**Table 3:** Multivariate Logistic regression with selected covariates measuring the association between primary delay and overall poor stage risk

**Variable**	**Odds ratio**	**Std. Err.**	**P > |0.05|**	**[95% Conf. Interval]**
Primary delay	3.41	1.36	0.002	1.46 - 7.45
*Selected covariates*				
Gender	0.50	0.23	0.144	0.19 - 1.27
Age	1.64	0.50	0.102	0.91 - 2.97
Income	1.00	0.35	0.992	0.51 - 1.99
Exposure to ART	0.60	0.25	0.216	0.26 - 1.35

In addition to primary delay, data were collected on the secondary delay and tertiary delay that was experienced by participants. Forty-seven (29%) patients experienced secondary delay longer than one month, while 26 (16%) experienced delay less than one week. Twenty-one (13%) patients experienced tertiary delay greater than 90 days, and the median delay time was 23 days (IQR: 11–47) with a mean delay of 42.7 days (SD: 53.1) (Table 4). Applying a cutoff of three weeks to define delay at both the secondary and tertiary stages revealed that 89% of the cohort experienced at least one type of delay, and 8% experienced delay at every stage from first noticing symptoms until diagnosis.

**Table 4:** Primary, secondary, and tertiary delay (n = 161)

**Delay type**		
	**Total**	
	**n**	**%**
**Primary delay**		
<3 Months	88	54.7
≥ 3 Months	73	45.3
**Secondary delay**		
>1 Week	26	16.1
2 Weeks - 1 Month	88	54.7
>1 Month	47	29.2
**Tertiary delay**		
0-7 Days	20	12.4
8-30 Days	73	45.3
31-90 Days	47	29.2
>90 Days	21	13.0

**Figure 2:**
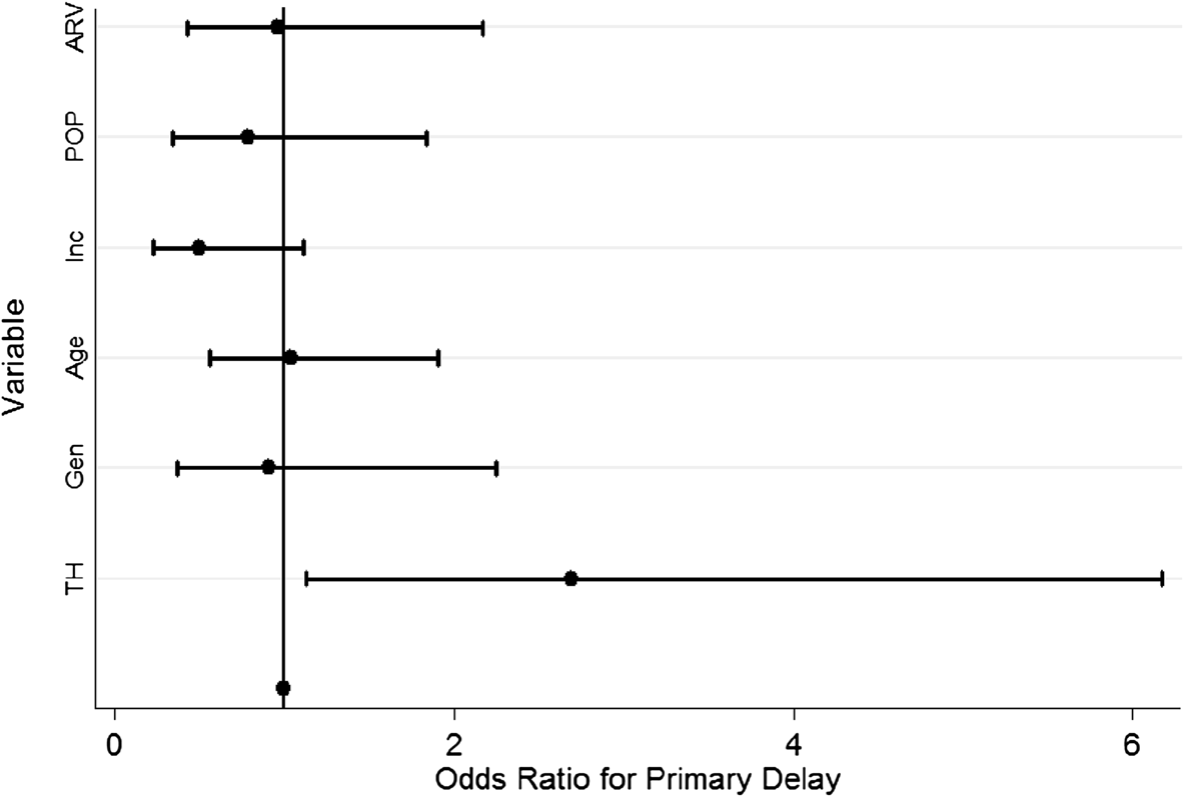
Plot of Odds Ratios for the association between visitation to a traditional healer and primary delay with selected covariates.

Multivariate analyses were also performed to measure associations between patient characteristics and experiencing diagnostic delay. After adjusting for gender, age, income, ability to pay out-of-pocket, and previous HIV clinic attendance, only visitation to a traditional healer was associated with experiencing diagnostic delay (OR 2.69, p = 0.020, 95% CI: 1.17-6.17). Previous HIV clinic attendance and duration on ART were not associated with delay (Figure 2, Table 5).

**Table 5:** Multivariate logistic regression with selected covariates measuring the association between visitation to a traditional healer and primary delay

**Variable**	**Odds ratio**	**Std. Err.**	**P > |0.05|**	**[95% Conf. Interval]**
Visitation to traditional healer	2.69	1.14	0.021	1.17 – 6.17
*Selected Covariates*				
Gender	0.91	0.42	0.835	0.37 – 2.25
Age	1.03	0.32	0.909	0.56 – 1.19
Income	0.50	0.21	0.092	0.23 – 1.11
Pay out-of-pocket	0.96	0.40	0.923	0.43 – 2.17
Exposure to HAART	0.79	0.34	0.581	0.34 – 1.84

## Discussion

Early diagnosis of cancer is an aim of cancer care and control programs worldwide, especially among LMICs that are experiencing a burgeoning cancer burden. This is particularly true for cancer care for HIV-associated malignancies that can leverage current HIV programs to detect cancer faster. Measuring delay can be an important step in better understanding patient outcomes, particularly in the context of under-resourced health systems. While numerous studies have offered theoretical frameworks for understanding delay, few have measured these delays across the entire continuum from first notice of signs and symptoms until treatment [18,24]. Moreover, to the best of our knowledge, none have examined delays and their influence on cancer stage or patient outcomes in resource-poor areas or delays with respect to HIV-associated malignancies in any context.

In response to this gap in understanding, this study measured the association between primary delay and the cancer stage upon admission among Kaposi’s sarcoma patients in Uganda. Our findings suggest, first, an association between primary delay and the KS cancer stage upon admission to a cancer referral center, and second, that delay can be a key point of intervention to improve overall KS stage risk. In this study, HIV-infected KS patients who experienced primary delay were more than three times as likely to have an overall poor stage risk at presentation compared those who did not experience primary delay. It is still unclear, however, how delay for KS patients and the effect of these delays in Uganda compares to other cancers given the dearth of data and inadequacy of comparing studies assessing cancer in high-income countries only [25-27].

Our study found far more patients (92%) presented with poor risk KS in the tumor extent category than in other studies, which suggest approximately 64% present with poor-risk disease [22,28]. However, these studies also lack comparative power since they evaluated patients in high-income settings. Later stage at diagnosis in our study is most likely reflective of diagnostic delay related to health system factors, especially concerning the role of traditional healers and the need for improvement among HIV health workers to recognize KS symptoms, as the majority of the cohort was previously exposed to HIV care. Much of the success in treating KS in high-income countries has been attributed to effective HIV referral systems that recognize KS signs and symptoms, along with widely available ART [26,27,29-31].

Rates of secondary and tertiary delays were lower in this cohort than in other studies [24,28]. These findings suggest that once primary delay is overcome, the health system at UCI performed better than those in comparable studies with respect to minimizing secondary and tertiary delay. These results should be interpreted with caution, however, as a large majority of the cohort (89%) experienced at least one type of delay and 8% experienced delay at every stage, leading to additive, lengthy delays across the care continuum. Further work should be done to examine the causes for lengthy delays at each point of the care continuum and role of the health system in creating delays.

The only patient characteristic associated with diagnostic delay in multivariate analysis was visitation to a traditional healer, which has been acknowledged in the literature as a competing source of healthcare and as a possible marker of poorer outcomes for cancer patients [29]. Traditional healers remain an important part of Ugandan societal norms, and results from this study suggest that working with traditional healers might present an important point of intervention to reduce delay.

Other results reveal an important opportunity for early KS detection that may be overlooked. A large proportion of this cohort was already exposed to the healthcare system via HIV care prior to admission to UCI. Though many patients developed signs and symptoms while seeking HIV care, they still experienced primary delay. As those patients enrolled in HIV care were no less likely to delay, this may present another opportunity for intervention. HIV/AIDS treatment programs have strategically leveraged their care infrastructure to target non-communicable diseases, but our data suggest more work needs to be done to ensure appropriate screening in this at-risk population [31]. Our study was not designed or powered to examine the relationship between HIV care and delay, but we did find that 93% of patients received some sort of treatment at an HIV clinic before admission to the UCI and that at least 41 patients (25%) were actually in HIV care when they developed signs and symptoms of KS, and 15 (37%) of these patients waited longer than three months before presenting to a clinician for those symptoms. Our study was not designed to analyze this finding that came out of our study, and more research is needed to examine the influence of previous HIV care on delay among KS patients in Uganda.

Our study is subject to limitations. Possible sources of bias include recall bias in determining delay and selection bias by interviewing patients only at a central referral center, potentially missing those were severely ill or those who improved on ART alone in their local clinic. The study only included out-patients, which may have led to an underrepresentation of severely ill patients. In addition, this represents one of the first studies implementing the ACTG staging system in a low-income setting with a population exposed to ART, and previous literature has shown that modification in staging for this population is necessary [23]. More research on the staging in the post-ART area is needed not only for KS, but other cancers as well [22,23,28,31].

In conclusion, reducing delay can be an important point of intervention for improving KS outcomes in Uganda. This is especially true for AIDS-associated malignancies with faster progression. In addition, leveraging traditional healers and pre-existing HIV referral system infrastructure to screen for cancer may be possible solutions. Finally, further investigations should examine the efficacy of KS staging in low-income settings of high ART exposure. While significant challenges exist for KS control in Uganda, these findings identify key interventions that can target inefficiencies in the health system and increase local cancer knowledge for faster KS referrals and better patient prognoses.

## Consent

Written informed consent was obtained from the patient for the publication of this report and any accompanying images.

## Competing interests

The authors declare that they have no competing interests.

## Authors’ contributions

CD and YZ were responsible for writing the manuscript. CD, YZ, and NN were involved in the analysis of data. All authors were involved in the study design, reviewing, and approving the manuscript.

## References

[B1] Ferlay J Soerjomataram I Ervik M Dikshit R Eser S Mathers C Rebelo M Parkin DM Forman D Bray F GLOBOCAN 2012 v1.0, Cancer Incidence and Mortality Worldwide: IARC CancerBase No. 11 [Internet] 2013 Lyon, France: International Agency for Research on Cancer Available from: http://globocan.iarc.fr/Default.aspx, accessed on 12/26/2013

[B2] Wabinga HR Nambooze S Amulen PM Okello C Mbus L Parkin DM Trends in the incidence of cancer in Kampala, Uganda 1991–2010 Int J Cancer 2013 doi:10.1002/ijc.28661 10.1002/ijc.28661 24615279

[B3] Chaabna K Bray F Wabinga HR Cokunonga E Borok M Vanhems P Soerjomataram I Kaposi sarcoma trends in Uganda and Zimbabwe: a sustained decline in incidence Int J Cancer 2013 133 5 1197 1203 10.1002/ijc.28125 23436712

[B4] Paparizos VA Kyriakis KP Papastamopoulos V Hadjivassiliou M Stavrianeas NG Response of AIDS-associated Kaposi sarcoma to highly active antiretroviral therapy alone J Acquir Immune Defic Syndr 2002 30 257 258 10.1097/00042560-200206010-00015 12045689

[B5] Dupin N de Cervens RV Gorin I The influence of highly active antiretroviral therapy on AIDS-associated Kaposi’s sarcoma Br J Dermatol 1999 140 875 881 10.1046/j.1365-2133.1999.02818.x 10354025

[B6] Wit FW Sol CJ Renwick N Roos MT Pals ST van Leeuwen R Goudsmit J Reiss P Regression of AIDS-related Kaposis sarcoma associated with clearance of human herpesvirus-8 from peripheral blood mononuclear cells following initiation of antiretroviral therapy AIDS 1998 12 218 219 9468373

[B7] Robles R Lugo D Gee L Jacobson MA Effect of antiviral drugs used to treat cytomegalovirus end-organ disease on subsequent course of previously diagnosed Kaposi’s sarcoma in patients with AIDS J Acquir Immune Defic Syndr Hum Retrovirol 1999 20 34 38 10.1097/00042560-199901010-00005 9928727

[B8] Winceslaus J Regression of AIDS-related pleural effusion with HAART: highly active antiretroviral therapy Int J STD AIDS 1999 9 6 368 370 9671256

[B9] Eltom M Jemal A Mbulaiteye S Trends in Kaposi’s sarcoma and non-Hodgkin’s lymphoma incidence in the United States from 1973 through 1998 J Natl Cancer Inst 2002 94 16 1204 1210 10.1093/jnci/94.16.1204 12189223

[B10] Nsubuga M Biggar R Combs S Marshall V Mbisa G Kambugu F Mehta M Biryahwaho B Rabkin CS Whitby D Mbulaiteye SM Human herpesvirus 8 load and progression of AIDS-related Kaposi sarcoma lesions Cancer Lett 2008 263 2 182 188 10.1016/j.canlet.2007.12.025 18234418 PMC2440724

[B11] Phipps W Ssewankambo F Nguyen H Saracino M Wald A Lawrence C Orem J Kambugu A Casper C Gender differences in clinical presentation and outcomes of epidemic Kaposi sarcoma in Uganda PLoS ONE 2011 5 11 e13936 doi:10.1371/journal.pone.0013936 21103057 10.1371/journal.pone.0013936 PMC2980479

[B12] Nguyen HQ Magaret AS Kitahata MM Van Rompaey SE Wald A Casper C Persistent Kaposi sarcoma in the era of highly active antiretroviral therapy: characterizing the predictors of clinical response AIDS 2008 22 937 945 10.1097/QAD.0b013e3282ff6275 18453853 PMC2730951

[B13] Robinson E Mohilever J Zidan J Sapir D Delay in diagnosis of cancer: possible effects on the stage of disease and survival Cancer 1984 54 1454 1460 10.1002/1097-0142(19841001)54:7<1454::AID-CNCR2820540739>3.0.CO;2-A 6467169

[B14] Porta M Gallen M Malats N Planas J Influence of diagnostic delay upon cancer survival: an analysis of five tumour sites J Epi Comm Health 1991 45 225 230 10.1136/jech.45.3.225 PMC1060763 1757766

[B15] Richards MA Westcombe AM Littlejohns P Ramirez AJ Influence of delay on survival in patients with breast cancer: a systematic review Lancet 1999 353 1119 1126 10.1016/S0140-6736(99)02143-1 10209974

[B16] Leidberg F Anderson H Mansson W Treatment delay and prognosis in invasive bladder cancer J Urol 2005 174 1777 1781 10.1097/01.ju.0000177521.72678.61 16217282

[B17] Ramos M Esteva M Cabeza E Campillo C Llobera J Aquilo A Relationship of diagnostic and therapeutic delay with survival in colorectal cancer: A review Eur J Cancer 2007 43 2467 2478 10.1016/j.ejca.2007.08.023 17931854

[B18] Goldsen RK Gerhardt PR Handy VH Some factors related to patient delay in seeking diagnosis for cancer symptoms Cancer 1957 10 1 1 7 10.1002/1097-0142(195701/02)10:1<1::AID-CNCR2820100102>3.0.CO;2-Z 13413795

[B19] Korsgaard M Pedersen L Laurberg S Delay of diagnosis and treatment of colorectal cancer—a population-based Danish study Cancer Detect Prev 2008 32 45 51 10.1016/j.cdp.2008.01.001 18406067

[B20] Hosseini SN Mousavinasab SN Moghimi MH Fallah R Delay in diagnosis and treatment of gastric cancer: from the beginning of symptoms to surgery—an andomi study Turk J Gastroenterol 2007 18 77 81 17602354

[B21] Krown SE Metroka C Wernz JC Kaposi’s sarcoma in the acquired immune deficiency syndrome: a proposal for uniform evaluation, response, and staging criteria. AIDS Clinical Trials Group Oncology Committee J Clin Oncol 1989 7 1201 1207 2671281 10.1200/JCO.1989.7.9.1201

[B22] Krown SE Testa MA Huang J AIDS-related Kaposi's sarcoma: prospective validation of the AIDS Clinical Trials Group staging classification. AIDS Clinical Trials Group Oncology Committee J Clin Oncol 1997 15 3085 3092 9294471 10.1200/JCO.1997.15.9.3085

[B23] Okuku F Orem J Kafeero J Phipps W Kamya MR Casper C Evaluation of the AIDS clinical trials group staging criteria for Kaposi sarcoma in a resource limited setting Infect Agents Canc 2012 7 suppl 1 P8. (19 April 2012)

[B24] Dwivedi A Dwivedi SN Suryanarayana D An epidemiological study on delay in treatment initiation of cancer patients Health 2012 4 2 66 79 10.4236/health.2012.42012

[B25] Salomaa ER Sallinen S Hiekkanen H Liipo K Delays in the diagnosis and treatment of lung cancer Chest 2005 128 2282 2288 10.1378/chest.128.4.2282 16236885

[B26] Abdel-Fattah MM Anwar MA Mar E Mari E El-Shazly MK Zaki AA Bedwani RN Nicolucci A Patient- and system-related diagnostic delay in breast cancer evidence from Alexandria, Egypt Eur J Pub Health 2009 9 15 19

[B27] Brower V AIDS-related cancers increase in Africa J Natl Cancer Inst 2011 103 12 918 919 10.1093/jnci/djr235 21693755

[B28] Nasti G Talamini R Antinori A AIDS-related Kaposi’s sarcoma: evaluation of potential new prognostic factors and assessment of the AIDS Clinical Trial Group staging system in the haart era—the Italian cooperative group on AIDS and tumors and the Italian cohort of patients naïve from antiretrovirals J Clin Onc 2003 21 2876 2882 10.1200/JCO.2003.10.162 12885804

[B29] Dye TD Bogale S Hobden C Complex care systems in developing countries Cancer 2010 116 3 577 585 10.1002/cncr.24776 20029968

[B30] UNAIDS Chronic care of HIV and noncommunicable diseases: How to leverage the HIV experience. UNAIDS REPORT 2011 [http://www.unaids.org/en/media/unaids/contentassets/documents/unaidspublication/2011/20110526_JC2145_Chronic_care_of_HIV.pdf]

[B31] Mbulaiteye S Bhatia K Adebamowo C Sasco AJ HIV and cancer in Africa: mutual collaboration between HIV and cancer programs may provide timely research and public health data Infect Agents Canc 2011 6 1 16 10.1186/1750-9378-6-16 PMC3223125 22004990

